# The Domestic Management of the Sick-Room, Necessary, in Aid of Medical Treatment, for the Cure of Diseases

**Published:** 1845-11

**Authors:** 


*The domestic management of the Sick-Room, necessary, in aid of medical treatment, for the cure of Diseases. By* ANTHONY TODD THOMPSON, M. D., F. L. S., etc. etc. *First American, from the second London edition. Revised, with additions,* by R. E. GRIFFITH, M. D., &c. pp. 353. Lea and Blanchard. Philadelphia, 1845.

This is a useful book, and particularly valuable to young physicians who are just entering upon the active duties of their profession. For nurses, too, and others who have occasion to attend upon the sick, provided they have intelligence enough to comprehend the directions it contains, (which is too seldom the case)

it will be found to contain much judicious instruction on all the points necessary for them to be informed. On a hasty perusal we have found nothing indeed to object to, except the portion of *Chapter VIII,* and "*Supplement,*" which treat of "domestic medicines." All attempts to popularize medicine, and especially by giving formulæ for the use of those unskilled in the science, in our apprehension, do harm. On no other subject are shallow draughts more apt to intoxicate the brain.

